# Enhanced photocatalytic efficiency of porous ZnO coral-like nanoplates for organic dye degradation†

**DOI:** 10.1039/d4ra01345j

**Published:** 2024-05-03

**Authors:** Nguyen Hong Hanh, Quan Thi Minh Nguyet, Tran Van Chinh, La Duc Duong, Tran Xuan Tien, Lai Van Duy, Nguyen Duc Hoa

**Affiliations:** a Institute of Engineering Physics, Academy of Military Science and Technology 17 Hoang Sam Street, Cau Giay District Hanoi City Vietnam hanh2904@gmail.com; b School of Engineering Physics, Hanoi University of Science and Technology (HUST) No. 1 Dai Co Viet Street Hanoi City Vietnam; c Institute of Chemistry and Materials, Academy of Military Science and Technology 17 Hoang Sam Street, Cau Giay District Hanoi City Vietnam; d Academy of Military Science and Technology 17 Hoang Sam Street, Cau Giay District Hanoi City Vietnam; e International Training Institute for Materials Science (ITIMS), Hanoi University of Science and Technology (HUST) No. 1, Dai Co Viet Street Hanoi Vietnam hoangduy141993@gmail.com duy.uniroma2@gmail.com; f Department of Food Quality and Nutrition, Research and Innovation Centre, Fondazione Edmund Mach 38010 San Michele all' Adige TN Italy; g Department of Electronic Engineering, University of Rome Tor Vergata 00133 Rome Italy; h Institute of Materials Science, Vietnam Academy of Science and Technology Hanoi City Vietnam

## Abstract

ZnO nanomaterials have been extensively used as photocatalysts for the removal of pollutants in aqueous environments. This study explores the enhanced photocatalytic performance of porous ZnO coral-like nanoplates synthesized *via* a one-pot wet-chemical method and subsequent annealing treatment. Characterization through scanning electron microscopy (SEM), high-resolution transmission electron microscopy (HRTEM), powder X-ray diffraction (XRD), energy-dispersive X-ray spectroscopy (EDX), Raman spectroscopy, photoluminescence (PL) spectroscopy, and Brunauer–Emmett–Teller (BET) measurements confirmed the nanoplates' porous structure, single-crystal structure, 100 nm thickness, and 80 nm pore size. These unique structural characteristics of the ZnO coral-like nanoplates enabled effective photodegradation of the organic dye rhodamine B (RhB) under visible light irradiation. Under simulated sunlight, the ZnO photocatalyst exhibited exceptional performance, achieving a 97.3% removal rate of RhB after 210 minutes of irradiation. The prepared ZnO photocatalyst also showed remarkable photostability and regeneration capability for RhB photodegradation with a decreased efficiency of less than 15% after eight testing cycles. The potential mechanism of the ZnO photocatalyst toward RhB degradation was also studied and is discussed in detail.

## Abstract 2

ZnO nanomaterials have been extensively used as photocatalysts for the removal of pollutants in aqueous environments.

## Graphical abstract

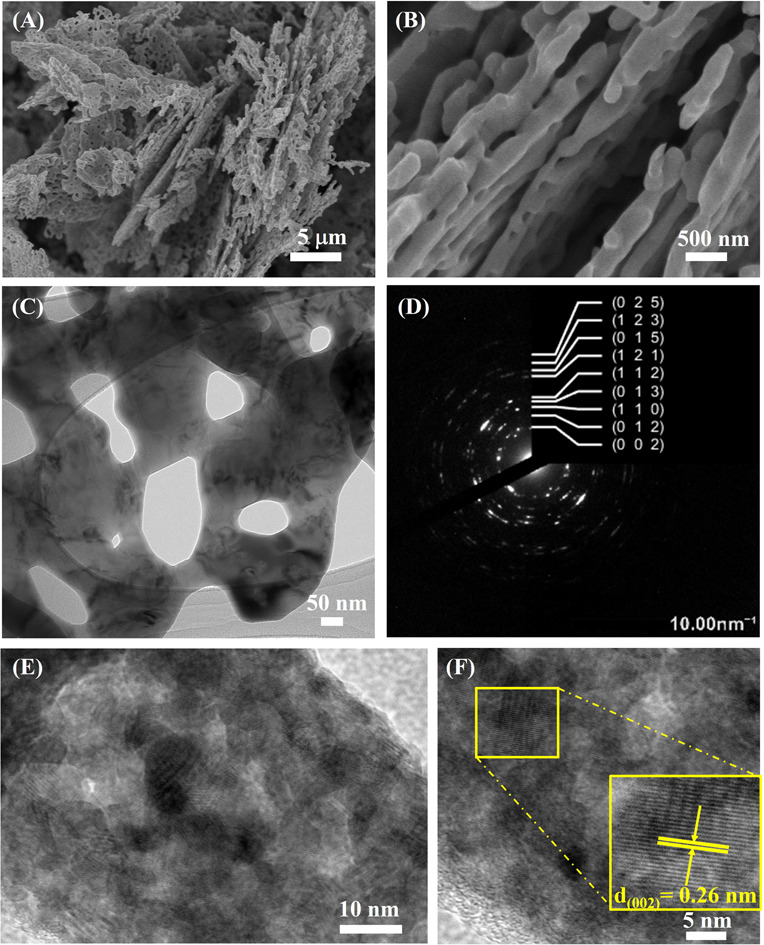

## Introduction

Industrial wastewater, being nonbiodegradable, highly toxic, and carcinogenic, with the disposal of partially or untreated wastewaters risks harm to human health and the environment. The industrial revolution, while propelling economic growth and technological strides, has also wrought environmental havoc. Among the prime culprits is the textile industry, a vital cog in the global economy. Together with sectors like textiles, pharmaceuticals, food, mining, paper industries, painting, printing, and leather, it releases a gamut of stubborn organic pollutants into the environment—dyes, pigments, phenols, pesticides, antibiotics, and more.^1,2^

The textile industry generates dye-contaminated wastewater, which elicits significant environmental concerns. Such wastewater contains intricate coloring compounds and persistent organic substances, such as azo derivatives, phenothiazines, and triphenylmethane.^3^ Specific examples include 4-nitrophenol (4-NP), naproxen (NPX), and rhodamine B (RhB).^4,5^ RhB, in particular, is recognized for its non-biodegradable, water-soluble and highly toxic nature. Even at concentrations below 1 ppm, RhB poses a severe threat to water quality, aquatic life, and human health. The removal of RhB from wastewater is crucial to mitigate its adverse impact on human health and the environment.^6^

Various techniques have been used to treat organic wastewater, but traditional methods often fall short in completely removing complex substances, leading to the persistence of non-biodegradable contaminants and requiring costly additional treatment. To address this, new strategies are urgently needed.^7^

Advanced oxidation, particularly photocatalysis, stands out as a promising and cost-effective method for removing RhB dye. Photocatalytic oxidation is preferred for its effectiveness, renewability, lack of secondary pollution, cost-effectiveness, and environmental friendliness. It surpasses conventional methods, making it the preferred choice for decomposing organic wastewater contaminants.^8,9^

Various nano-sized photocatalysts, such as Ag, Ag_3_PO_4_, CdS, CuO, V_2_O_5_, TiO_2_, g-C_3_N_4_, ZnO, porphyrin, AB_2_X_4_, and their combinations, have proven effective in photocatalytic treatment processes.^10–12^

ZnO stands out among them due to its easy synthesis, low cost, ready availability, high chemical and thermal stability, non-toxicity, and environmental friendliness. Its wide bandgap of 3.37 eV and high exciton binding energy of 60 meV at room temperature (300 K) make ZnO a highly efficient photocatalyst.^13^ ZnO nanomaterials exhibit exceptional electrical and optical properties, including piezoelectricity, pyroelectricity, luminescence, semiconductivity, and catalytic activity. Their applications span hydrogen production, gas sensors, biosensors, transistors, cosmetics, and solar cells.^14,15^

Recent research has focused on developing ZnO nanostructures with diverse morphologies (such as nanowires, nanobelts, nanoflakes, nanosheets, nanoparticles, nanoneedles, and flower-like structures) for effective RhB dye removal.^16^ Key factors influencing the photocatalytic performance of semiconductor materials include bandgap energy, morphology, specific surface area, crystallinity, crystal faces, nanoparticle size, and surface/bulk defects.^17,18^ The synthesis of ZnO nanostructures is pivotal in photocatalysis due to the substantial impact of morphology on performance. Significantly, the optical performance of two-dimensional (2D) porous ZnO nanomaterials is enhanced by their large specific surface area to volume ratio, offering a substantial quantity of active adsorption sites.^19^

For instance, ZnO nanostructures, comprising nanoparticles and flowers, have been successfully prepared using a facile hydrothermal method. Through the use of microwave assistance in hydrothermal synthesis and subsequent calcination at different temperatures, Mousavi *et al.* created porous ZnO nanostructures.^18^ The 700 °C calcinated ZnO exhibited optimal photocatalytic performance (>99%) under both UV light and sunlight, attributed to its porous structure facilitating efficient RhB degradation. The nanostructures maintained high efficiency through multiple reuse cycles. A study led by Aminah Umar *et al.*^20^ explored ZnO nanoparticle synthesis using *Sapindus rarak* DC fruit pericarp extract, highlighting heightened photocatalytic efficiency attributed to the smaller particle size of ZnO. The nanoparticles, with their unique surface structure, exhibited superior performance, achieving a remarkable 99.7% degradation of RhB within 120 minutes. The porous nanosheet structure morphology of ZnO plays a critical role in enhancing the decomposition of organic dyes by facilitating visible-light-driven dye-photosensitized degradation. This structural characteristic, exemplified in the study by Xiaohua Sun *et al.*,^21^ underscores its pivotal role in catalyzing efficient dye decomposition processes.

D.-Y. Zhou *et al.*^22^ fabricated ZnO–nano ZnO@porous carbon (ZnO–nZnO@PC) using the Ostwald ripening mechanism and a simple pyrolysis process. The improved photocatalytic activity is attributed to several factors: tight binding facilitated by ZnO nanorods acting as sacrificial templates, controlled carbon layer thickness regulating ZnO encapsulation, enhanced specific surface area and adsorption capacity, facilitated charge carrier transportation, and boosted photocatalytic activity due to the piezoelectric effect induced by ultrasonic energy.

This study outlines a simple hydrothermal method for producing porous ZnO coral-like nanoplates with large pores, aiming at enhancing the photocatalytic removal of RhB dye in sunlight. By adjusting urea and zinc nitrate concentrations, we successfully controlled the morphology of the nanoplates. Notably, those synthesized at 600 °C exhibited outstanding photocatalytic performance, achieving over 99% degradation of RhB within 60 minutes under natural sunlight. The coral-like nanoplates, characterized by high crystallinity and surface defects, exhibit a porous structure formed from the decomposition of hydrozincite Zn_5_(CO_3_)_2_(OH)_6_ upon firing, releasing H_2_O and CO_2_. This network structure significantly enhances the material's accessible surface area, facilitating efficient organic dye decomposition as a catalytic material. Furthermore, we assessed the photostability and regeneration capability of these nanoplates through multiple reuse photocatalytic experiments. The results underscore a significant advancement in utilizing non-toxic and environmentally friendly oxide semiconductors for efficiently degrading water pollutants under natural sunlight irradiation.

## Results and discussion

**Fig. 1:**
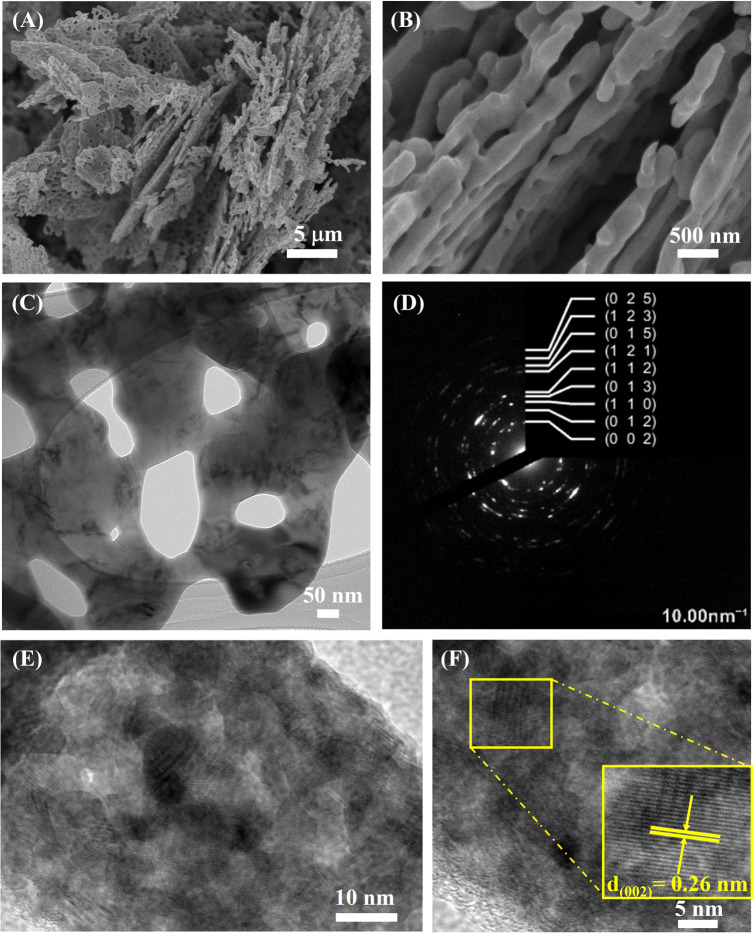
(A and B) SEM images and (C) low- and (E and F) high-magnification TEM images of porous ZnO coral-like nanoplates annealed at 600 °C for 2 h in air. (D) Corresponding selected area electron diffraction.

SEM images of ZnO coral-like nanoplates annealed at 600 °C for 2 hours are presented in Fig. 1(A) and (B).

The low-magnification image in Fig. 1(A) illustrates uniform coral-like nanoplates with persistent pores after heating. The high-magnification image in Fig. 1(B) reveals nanosized porous structures with edges measuring 30–50 nm. Calcination at 600 °C introduces jagged edges and broader 50–100 nm pores. The presence of small, evenly distributed 50–100 nm pores enhances the nanoplates' surface area, thereby boosting photocatalytic efficiency. The synthesis method avoids the use of surfactants, minimizing chemical consumption. Zinc nitrate hexahydrate serves as the Zn^2+^ precursor, and urea regulates pH, decomposing into NH_3_, NH_4_OH and HNCO during hydrothermal processing. The resulting reaction yields ZnO with the observed porous structure. Morphology and thickness are determined by the choice of precursor salt.

TEM characterization was utilized to delve deeper into the morphology and structure of the porous ZnO coral-like nanoplates. Fig. 1(C) presents a low-magnification TEM image of ZnO coral-shaped nanoplates, in which the nanoplates reach the micrometer scale in length with large nanopores of diameter of approximately 100 nm. Additionally, HRTEM images and respective SAED pattern which are shown in Fig. 1(D–F) present luminous rings with distinct spots, confirming the well-defined and polycrystalline nature of annealed ZnO nanosheets. The lattice plane of bright ring patterns was determined based on Crys TBox software and depicted pictorially in the inset figure, consisting of (002), (012), (110), (013), (112), (121), (015), (123), and (025) planes in Fig. 1(D). Moreover, the interplanar distance measured roughly 0.26 nm with respect to the (002) plane of hexagonal ZnO microstructure in Fig. 1(F), this being consistent with the previous analyzed result of the SAED pattern.

**Fig. 2:**
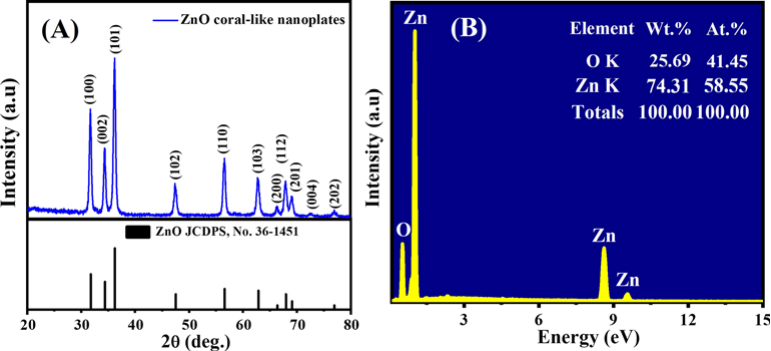
(A) XRD pattern and (B) EDS spectrum of the synthesized porous ZnO coral-like nanoplates annealed at 600 °C for 2 h in air.

Fig. 2(A) illustrates the XRD pattern of the coral-like nanoplates, confirming their crystalline nature as ZnO with a wurtzite structure (hexagonal ZnO, JCPDS No. 36-1451). The (100), (002), (101), (102), (110), (103), (200), (112), (201), (004), and (202) planes correspond to the observed diffraction peaks at 2*θ* values of 31.77°, 34.42°, 36.25°, 47.53°, 56.60°, 62.86°, 66.38°, 67.98°, 69.1°, 72.58°, and 76.95°, respectively. A single phase of ZnO is shown by the XRD pattern's lack of aberrant diffraction peaks. The porous coral-like nanoplates have a high degree of crystallization, as confirmed by sharp and intense peaks. The highest peak intensity clearly shows that (101) is the preferred growth direction. The calculated average crystallite size using the Scherer equation is approximately 17.45 nm. These findings highlight the well-defined structural characteristics of the synthetic porous ZnO coral-like nanoplates annealed at 600 °C for 2 h in air, emphasizing their high crystallinity and preferred growth orientation.

The EDS trace of the porous ZnO coral-like nanoplates is illustrated in Fig. 2(B). Through qualitative and quantitative EDS analyses, the atomic ratios were established at 58.55% for zinc and 41.45% for oxygen. The discernible deviation from stoichiometry suggests the existence of oxygen defects, classifying the material as an n-type semiconductor. The absence of impurities highlights the exceptional quality of the synthesized nanoplates.

**Fig. 3:**
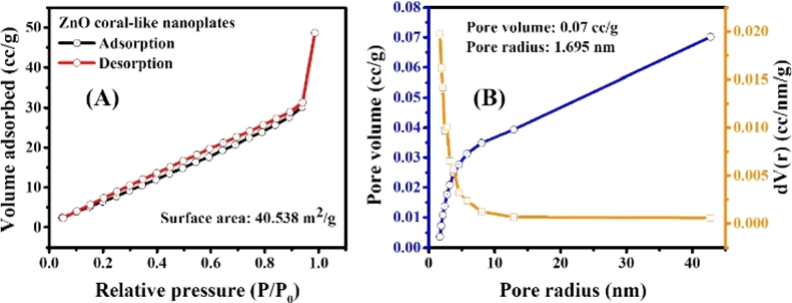
Nitrogen adsorption/desorption isotherm of (A) ZnO coral-like nanoplates, and (B) pore size distribution.

The surface area properties of a photocatalyst play a vital role in determining the productivity of photoreaction. Therefore, the BET surface area and pore size distribution of ZnO coral-like nanoplates were characterized by the N_2_ adsorption–desorption isotherm, as shown in Fig. 3. It is obvious that the ZnO nanoplates had a high surface area of 40.538 m^2^ g^−1^ with considerable pore volume and pore size of 0.07 cm^3^ g^−1^ and 1.695 nm, respectively. Despite the pronounced porosity exhibited by the ZnO coral-like nanoplates, their pore sizes are relatively small. This characteristic attribute precipitates a consequential reduction in active adsorption sites, thereby exerting a tangible influence on the photocatalytic performance of the material.

**Fig. 4:**
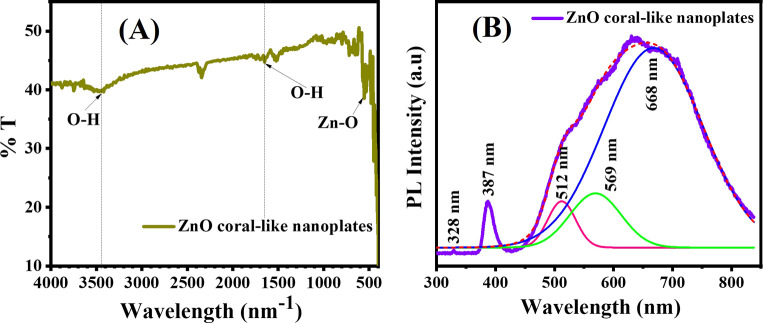
(A) FTIR spectrum and (B) PL spectrum of porous ZnO coral-like nanoplate material.

Fourier transform infrared (FTIR) analysis was employed to evaluate the chemical composition and identify functional groups in the prepared coral-like nanoplates (Fig. 4(A)). The spectrum of unaltered nanoplates exhibited a distinctive absorption band at 3424 cm^−1^, indicating the presence of the O–H vibrational mode. Peaks at 3426 and 1647 cm^−1^ were attributed to O–H stretching and bending vibrations of surface hydroxyl groups on ZnO. The prominent peak at 548 cm^−1^ corresponds to the stretching vibrations of Zn–O bonds, characteristic of the Zn–O stretching frequency in octahedral coordination.^23^

Fig. 4(B) depicts the photoluminescence (PL) spectrum of porous ZnO coral-like nanoplates excited at 325 nm using a helium–cadmium (He–Cd) laser. The PL spectrum results of ZnO, as described, indicate a complex emission profile attributed to various defects and vacancies within the material. Utilizing a multi-peak Gaussian fitting technique, the spectrum of the ZnO sample was resolved into three emission bands: a broad peak centered around 387 nm, a weaker band spanning the visible range from 512 to 569 nm, and a third emission band at approximately 568 nm. The 387 nm UV peak signifies near-band-edge emission, indicative of band-to-band transitions within ZnO nanocrystals with an energy level of 3.188 eV. The reduced bandgap of the nanosheets, compared to bulk ZnO (3.37 eV), is attributed to optical confinement effects.^24,25^

The presence of oxygen vacancies within the ZnO lattice, highlighted by the pink band at approximately 512 nm in the second emission band (spanning 512–569 nm), is suggested. Oxygen vacancies introduce energy levels within ZnO's bandgap, resulting in visible-range emission bands. These vacancies significantly impact the material's photocatalytic properties by altering charge carrier dynamics and surface reactivity. Weaker peaks in the visible region are likely attributed to excitons and defect states on the porous ZnO coral-like nanoplates' surface. This underscores the substantial normalized PL intensity observed for the coral-like porous ZnO nanosheet sample, consistent with pure n-type semiconductor properties, distinguishing it from previous reports involving precious metal-decorated samples.^26^

Additionally, the third emission band at around 568 nm, attributed to a blue band indicative of Zn vacancies, reveals their presence within the ZnO crystal structure. Similar to oxygen vacancies, zinc vacancies introduce defect levels in the bandgap, affecting the material's electronic structure and optical properties.

**Fig. 5:**
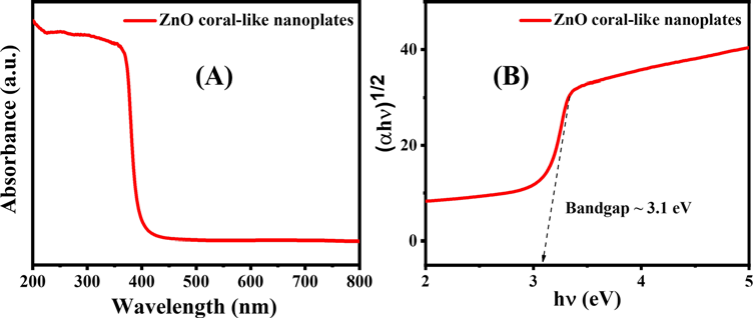
(A) UV-visible spectrum for porous ZnO coral-like nanoplate samples. (B) Corresponding Tauc plot and estimated bandgap of the synthesized material.

Fig. 5(A and B) depict the bandgap analysis of porous ZnO coral-like nanoplates using Tauc's formula. The formula establishes a relationship between absorption coefficient (*α*) and photon energy (*hν*) as:1
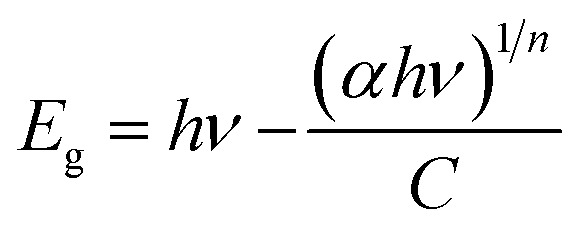
where *n* represents the bandgap exponent, and Eg is the bandgap energy, with a fixed value of 2 for ZnO's direct bandgap. Extrapolating the linear portion of the (*αhν*)^1/*n*^*versus hν* graph provides the bandgap values. In the case of pure porous ZnO coral-like nanoplates, the obtained bandgap is 3.1 eV. This finding closely aligns with earlier research, confirming the consistency of results.

**Fig. 6:**
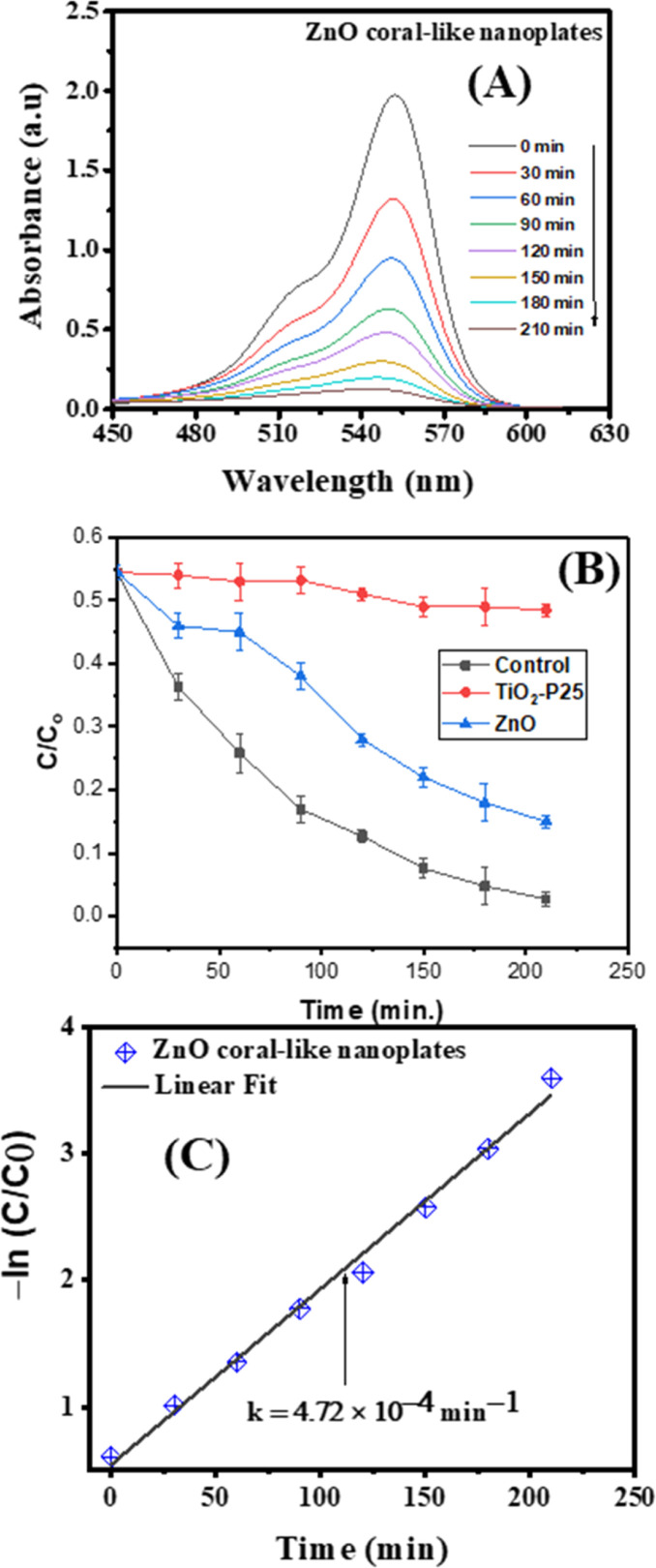
(A) The time-dependent efficiency of porous ZnO coral-like nanoplates in removing RhB. (B) Adsorption–photodegradation dynamics. (C) Results of kinetics study under simulated sunlight for 210 minutes.

The photocatalytic efficiency of porous ZnO coral-like nanoplates was assessed in the decomposition of RhB dye (a representative modeling dye) compared to control and P25 (TiO_2_) photocatalyst. It can be clearly seen from Fig. 6(B) that the photocatalytic activity of the ZnO coral-like nanoplates is higher than that of commercial P25 catalyst. Conducted under simulated sunlight, the UV-visible light irradiation test monitored the decline in RhB concentration using a spectrophotometer. Over time, there was a notable reduction in the maximum absorption peak intensity of RhB (554 nm) when utilizing porous ZnO coral-like nanoplates as a photocatalyst, indicating superior activity (Fig. 6(A)). The experiment demonstrated a significant 97.3% degradation of RhB dye after 210 minutes (Fig. 6(B)), with a calculated RhB degradation rate of approximately −4.72 × 10^−4^ min^−1^.

A comparison between our current investigation utilizing a ZnO catalyst under visible light irradiation and the existing body of literature reveals significant complexity. Numerous researchers have explored the photodegradation of RhB using various ZnO structures and hybrid compositions incorporating ZnO alongside other semiconducting metal oxides (Table 1). However, direct comparison of their photocatalytic performances proves challenging due to the absence of standardization across various parameters such as fabrication methods, reaction conditions, reactor setups, and light sources.

**Table 1:** Comparison of photodegradation of rhodamine B using various ZnO structures and hybrid compositions incorporating ZnO alongside other semiconducting metal oxides

Photocatalyst	Weight of catalyst	Volume RhB of solution	Initial RhB solution conc.	Photodegradation efficiency	Ref.
ZnO nanoparticles	2 g L^−1^	—	25 mg L^−1^	95%, 70 min	14
Ag@ZnO	—	200 mL	20 mg L^−1^	95%, 120 min	27
Undoped ZnO	5 mg	20 mL	4 mg L^−1^	81%, 120 min	28
Ce–ZnO nanoparticles	80 mg	80 mL	10^−5^ mol L^−1^	97.72%, 120 min	29
Ag@ZnO	0.03 mg	100 mL	10 mg L^−1^	91%, 180 min	3
Ag/ZnO@N-carbon	50 mg	100 mL	5 ppm	98.65%, 25 min	30
Mo-doped ZnO 1%	500 mg	100 mL	5 mg L^−1^	95%, 60 min	23
Cu–ZnO/rGO nanorod-like film	100 mg	50 mL	5 ppm	95%, 420 min	26
AgNPs@ZnO composite	50 mg	50 mL	10 mg L^−1^	95%, 180 min	31
g-C_3_N_4_/α-Fe_2_O_3_	50 mg	100 mL	10 mg L^−1^	97.2%, 150 min	32
Fe_2_O_3_/g-C_3_N_4_	0.1 g L^−1^	10 mL	5 mg L^−1^	94.8%, 140 min	33
Fe_3_O_4_/ZnO/g-C_3_N_4_	50 mg	60 mL	20 mg L^−1^	92%, 150 min	34
TiO_2_ (P25)	25 mg	—	10 mg L^−1^	83.5%, 240 min	35
TiO_2_/VA-CNT	—	20 mL	5 mg L^−1^	55%, 240 min	36
ZnO	30 mg	20 mL	20 mg L^−1^	97.3%, 210 min	This work

Our study, employing simulated sunlight, demonstrates superior energy efficiency compared to conventional photocatalytic processes. Furthermore, the heightened crystallinity of the porous ZnO coral-like nanostructure notably enhances the photodegradation efficiency of RhB dye in water, outperforming previously reported photocatalysts such as P25 particles (TiO_2_) and TiO_2_-based hybrid structures (as illustrated in Table 1).

**Fig. 7:**
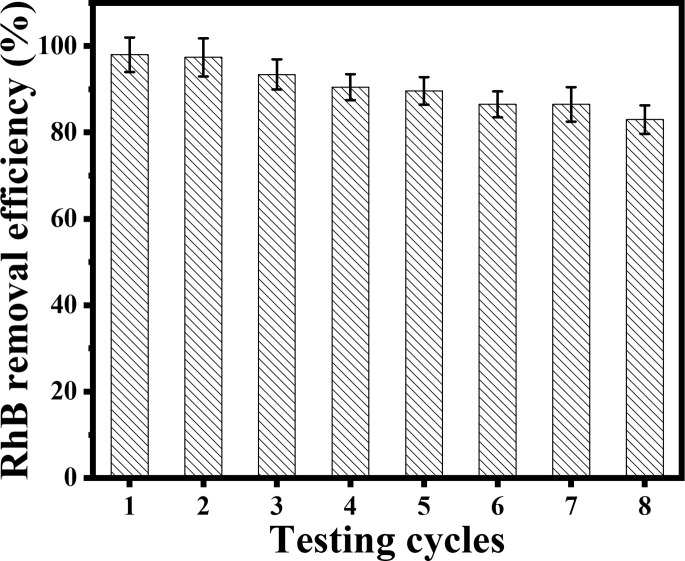
The stability of ZnO's photocatalytic activity for the degradation of RhB.

The stability of photocatalytic materials is important for practical application. As previously mentioned, the resulting porous ZnO coral-like nanoplates may be easily extracted from solution and repurposed for further RhB treatment cycles by centrifugation. The results of the recyclability study of the ZnO photocatalyst toward RhB under simulated sunlight irradiation are shown in Fig. 7. With a removal efficiency that drops from 98% for the first cycle to around 83% for the eighth cycle, it is evident that the composite's photocatalytic efficacy only slightly decreases after eight cycles. This finding implies that the ZnO coral-like nanoplates' photocatalytic activity is robust for practical application.

The ZnO photocatalyst after treatment of RhB was characterized using FTIR spectroscopy and the result is shown in Fig. S1.† It is obvious that no vibration bands of RhB or intermediates were observed in the FTIR spectrum. This means that the RhB compound was degraded and mineralized to form products such as CO_2_ and H_2_O.

The photocatalytic degradation of RhB by the porous ZnO coral-like nanoplates under simulated sunlight involves the absorption of photons near the semiconductor bandgap, generating electron–hole (e^−^/h^+^) pairs.

**Fig. 8:**
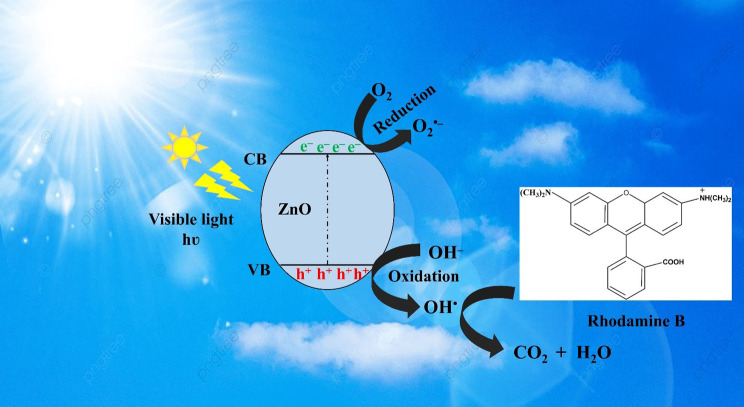
Photocatalytic degradation mechanism of RhB by porous ZnO coral-like nanoplates under simulated sunlight.

The photodegradation of the RhB organic dye by the ZnO coral-like nanoplates is shown in Fig. 8. The porous ZnO coral-like nanoplates absorb photons with energies near to the semiconductor bandgap when they are subjected to simulated sunlight. Within the semiconductor, electron–hole (e^−^/h^+^) pairs are produced as a result of this absorption.^37^ Either recombination or migration of the photoinduced electron–hole pairs to the semiconductor surface is possible. The photoinduced electron–hole pairs can either recombine or migrate to the semiconductor surface. Migration allows the separated electrons (e^−^) and holes (h^+^) to participate in subsequent reactions. Once at the semiconductor surface, the separated electrons and holes engage in a series of oxidation–reduction reactions with adsorbed species on the nanoplate surface. This includes reactions with organic dyes like RhB that are adsorbed onto the catalyst. Highly reactive hydroxyl radicals (˙OH) are created when the photogenerated holes in the valence band react with adsorbed water molecules and surface-bound hydroxyl groups (–OH). O_2_˙^−^, or superoxide, radicals are created when electrons in the conduction band combine with oxygen molecules.^38^ The O_2_˙^−^ species contribute to the formation of hydrogen peroxide (H_2_O_2_), which, combined with the ˙OH radicals formed earlier, plays a crucial role in the degradation of the organic dye. The resulting reactive species, including hydrogen peroxide (H_2_O_2_), effectively oxidize and degrade RhB, yielding simpler, less harmful compounds.

Porous ZnO coral-like nanoplates' specific surface area, bandgap energy, shape, crystallinity, crystal facets, surface and bulk defects, and nanoparticle size all affect the photocatalytic performance. Surprisingly, porous ZnO nanostructures, calcined at 600 °C, exhibited a relatively high degradation rate despite having the lowest specific surface area.^39^

The key determinant impacting their photocatalytic activity was identified as the nanostructures' crystallinity. It was discovered that a decline in the proportional concentration of bulk to surface flaws was associated with high crystallinity. The adsorption sites and charge carrier traps that surface imperfections provide are essential in stopping the recombination of photogenerated electron–hole pairs. Bulk flaws, on the other hand, serve as recombination sites and reduce photocatalytic activity.

The tradeoff that has been seen in ZnO nanostructures between specific surface area and the concentration ratio of surface to bulk defects highlights the importance of high crystallinity in controlling the photocatalytic effectiveness.

## Experimental

### Materials

Urea (CH_4_N_2_O) and Zn(NO_3_)_2_·6H_2_O were purchased from Sigma-Aldrich (St. Louis, MO, USA) and ethanol (CH_3_CH_2_OH, 99.8%) was obtained from Xilong Chemicals (China). All reagents, of analytical grade, were used without purification. Deionized water served as the solvent for solution preparation.

### Fabrication of ZnO coral-like nanoplates

Porous ZnO coral-like nanoplates were successfully synthesized *via* a hydrothermal method, employing urea as an intermediate to regulate pH, zinc nitrate as the precursor, and deionized water as the medium, as detailed in prior studies.^13,40^

Typically, zinc nitrate served as the precursor in 30 mL of a deionized water solution, stirred for 15 min. Subsequently, 20 mL of urea solution (8 mmol) was added and stirred for an additional 15 min until reaching a pH of 5. The resultant mixture was put through a 24 hours hydrothermal procedure at 220 °C in a 100 mL stainless-steel autoclave lined with Teflon.

The precipitate was centrifuged, cooled gradually to room temperature, and then twice washed with ethanol solution and deionized water. For a full day, the gathered white powder was dried at 60 °C in an oven. Finally, the product underwent calcination at 600 °C to complete the synthesis process.

### Characterization techniques

The crystallinity was analyzed using XRD with a Cu Kα source (X-Pert Pro, Malvern Panalytical Ltd, Malvern, UK) in a 2*θ* range of 10° to 80°. Energy-dispersive analysis was performed with a HORIBA system (Minami-ku Kyoto, Japan). Vibrational energy levels were studied through Raman spectroscopy (InVia confocal micro-Raman spectroscope, Renishaw, UK). SEM (FESEM, Hitachi S-4800, Tokyo, Japan) provided morphology images of the porous ZnO coral-like nanoplates, while HRTEM (JEM 2100, JEOL Ltd, Tokyo, Japan) offered high-resolution microstructure insights. Optical properties were assessed *via* PL measurements at 25 °C with an excitation wavelength of 325 nm. Using BET analysis (Micromeritics Gemini VII 2390, Micromeritics Instrument Corporation, Norcross, GA, USA), specific surface area was ascertained. UV-visible spectroscopy (Shanghai Yoke Instrument Co. Ltd, China) was employed to study the optical properties of the materials. Additionally, the photocatalytic activity of the porous ZnO coral-like nanoplates in decomposing RhB dye was investigated using a UV-visible spectrophotometer.

### Photocatalytic experiments

The photocatalytic efficiency of ZnO in decomposing RhB in aqueous media was assessed. Following a standard experiment, a 20 ppm RhB stock solution (20 mL) was placed in a 25 mL vial, and 30 mg of ZnO catalyst was added. This mixture equilibrated overnight in the dark to establish adsorption/desorption equilibrium before undergoing irradiation. The photodegradation performance of ZnO in removing simulated pollutants, specifically the organic dye RhB, was investigated under simulated sunlight generated by a 350 W air-cooled xenon lamp (from China, 350 W). The entire experiment was conducted within an isolated reaction chamber. At 30 minutes intervals, approximately 3 mL of the RhB solution was withdrawn. The extraction solution was then centrifuged at 4000 rpm for 1 minute to remove all ZnO catalyst. The resulting solution was analyzed using a UV-visible spectrophotometer, and the RhB concentration was determined at the maximum absorption wavelength (*λ*_max_) of 553 nm. This process allowed continuous monitoring of RhB degradation over time in the presence of the nanostructured ZnO photocatalyst.

## Conclusions

In conclusion, this study demonstrates a facile hydrothermal method for synthesizing porous ZnO coral-like nanoplates with large pores, tailoring their morphology by adjusting urea and zinc nitrate concentrations. The optimized conditions at 600 °C resulted in nanoplates exhibiting exceptional photocatalytic performance, achieving over 97.3% degradation of RhB dye within 210 minutes under natural sunlight. The efficacy is attributed to their high crystallinity and proportion of surface defects. This outstanding efficiency is attributed to the plate nanoporous structure and inherent defects. The identification of predominant active species, ˙O_2_^−^ and OH^·^, coupled with an analysis of kinetic factors, enhances our understanding of the photodegradation mechanism. Moreover, the photostability and regeneration capability of these nanoplates were evaluated through multiple reuse photocatalytic experiments, showcasing their potential for sustainable water pollutant degradation under sunlight irradiation. The findings underscore the potential of these nanostructures for advanced applications in environmental remediation, emphasizing their significance in harnessing solar energy for efficient organic dye degradation. This research contributes valuable insights into the design and optimization of ZnO nanostructures for enhanced photocatalytic applications.

## Author contributions

N. H. Hanh, T. V. Chinh: investigation, data collection, writing – original draft preparation. Q. T. M. Nguyet, T. X. Tien, L. V. Duy: resources, reviewing and editing; N. D. Hoa, L. D. Duong: writing – reviewing and editing. N. D. Hoa: visualization, editing, funding acquisition & supervision. All authors approved the manuscript.

## Conflicts of interest

The authors declare that they have no known competing financial interests or personal relationships that could have appeared to influence the work reported in this paper.

## Supplementary Material

RA-014-D4RA01345J-s001
