# Supplementary material for: Enhanced photocatalytic efficiency of porous ZnO coral-like nanoplates for organic dye degradation
Source: RSC Adv. 2024 May 3;14(21):14672–9. doi: 10.1039/d4ra01345j (PMC11067434; doi:10.1039/d4ra01345j)
Supplement: RA-014-D4RA01345J-s001 [file RA-014-D4RA01345J-s001.pdf]

Electronic Supplementary Material (ESI) for RSC Advances.
This journal is © The Royal Society of Chemistry 2024

# Supporting Information

## Enhanced Photocatalytic Efficiency of Porous ZnO Coral-like Nanoplates for Organic Dye Degradation

Nguyen Hong Hanh, *[a] Quan Thi Minh Nguyet,[b] Tran Van Chinh, [c] La Duc Duong, [c] Tran Xuan Tien, [d] Lai Van Duy, *[e, f, g] Nguyen Duc Hoa[e]

[a] *Institute of Engineering Physics, 17 Hoang Sam Street, Cau Giay District, Hanoi City, Vietnam.*
[b] *School of Engineering Physics, Hanoi University of Science and Technology (HUST), No 1 Dai Co Viet Street, Hanoi City, Vietnam.*
[c] *Institute of Chemistry and Materials, Inorganic Chemistry, 17 Hoang Sam Street, Cau Giay District, Hanoi City, Vietnam.*
[d] *Academy of Military Science and Technology, 17 Hoang Sam Street, Cau Giay District, Hanoi City, Vietnam.*
[e] *International Training Institute for Materials Science (ITIMS), Hanoi University of Science and Technology (HUST), No 1, Dai Co Viet Street, Hanoi, Vietnam.*
[f] *Department of Food Quality and Nutrition, Research and Innovation Centre, Fondazione Edmund Mach, 38010, San Michele all' Adige, TN, Italy.*
[g] *Department of Electronic Engineering, University of Rome Tor Vergata, 00133 Rome, Italy.*

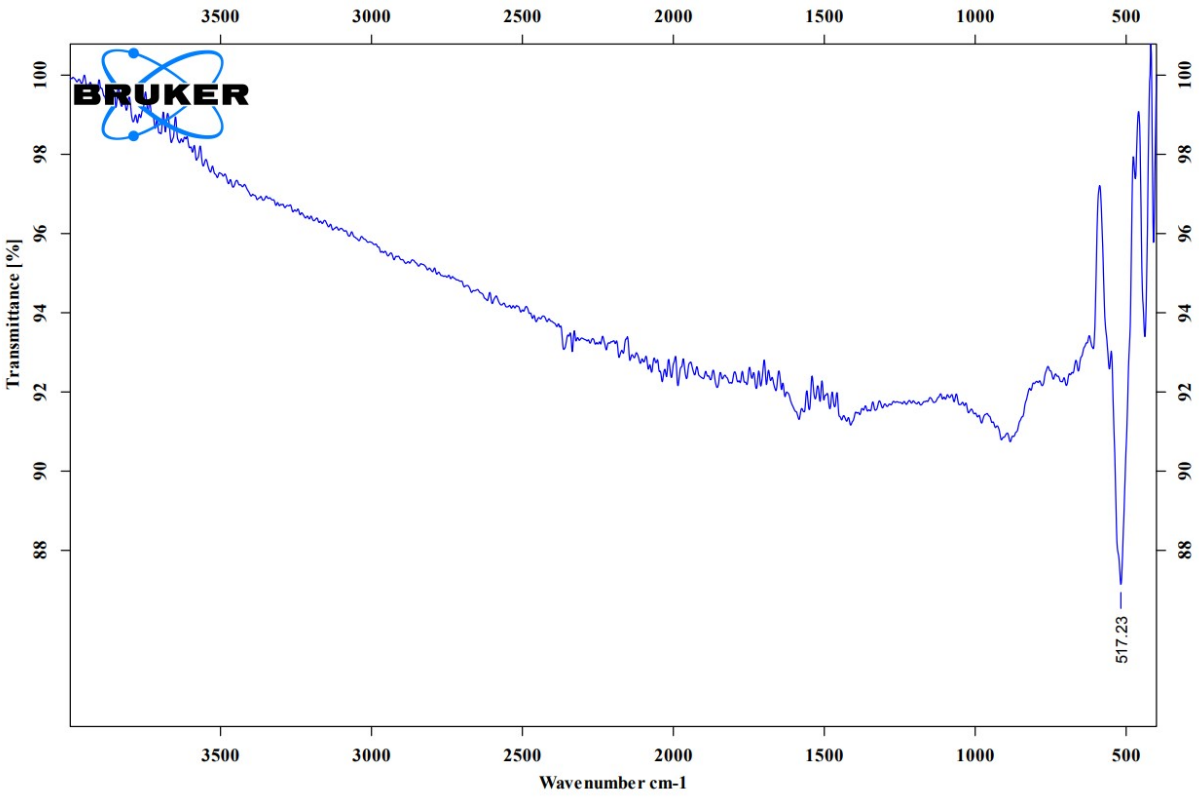


**Figure S1.** FTIR spectrum of spent ZnO nanoplates after used as photocatalyst for RhB degradation.
